# Intracardiac Migration of the Ureteral Double-J Stent during Percutaneous Nephrolithotomy

**DOI:** 10.3390/medicina57090939

**Published:** 2021-09-06

**Authors:** Chun-Kai Chang, Yi-Hsuan Wu, Ming-Chen Paul Shih, Jiun-Hung Geng

**Affiliations:** 1 Department of Urology, Kaohsiung Medical University Hospital, Kaohsiung 80756, Taiwan; kai05010501@gmail.com; 2 Department of Urology, Kaohsiung Municipal Siaogang Hospital, Kaohsiung 81267, Taiwan; maivy0314@gmail.com; 3 Department of Radiology, Kaohsiung Medical University Hospital, Kaohsiung 80756, Taiwan; mcshih@kmu.edu.tw; 4 Graduate Institute of Clinical Medicine, College of Medicine, Kaohsiung Medical University, Kaohsiung 80756, Taiwan

**Keywords:** complication, intracardiac migration, ureteral double-J stent, nephrolithiasis, percutaneous nephrolithotomy

## Abstract

The complications of percutaneous nephrolithotomy (PNL) include hemorrhage, damage to adjuvant organs, and other medical issues, although intracardiac migration of ureteral double-J stent has never been found during PNL and delaying the diagnosis might cause mortality. We report the case of a 60-year-old male who was admitted to receive one-stage PNL for right renal stones. During operation, an unexpected atrial fibrillation with a drop in blood pressure was suddenly encountered and the chest X-ray subsequently showed that the ureteral double-J had penetrated deep into the heart. Emergent endovascular intervention was performed to remove the stent and the patient was uneventfully discharged 2 days later.

## 1. Introduction

Despite the rapid development of retrograde intrarenal surgery, percutaneous nephrolithotomy (PNL) is still the standard procedure for large kidney stones [1,2]. PNL provides a safe and effective management for renal calculi [3,4] with acceptable complications [5]; however, major complications might cause mortality if not recognized in a timely fashion. Herein, we present a rare, first-described major complication concerning ureteral double-J stent migration penetrating into the heart during PNL and demonstrate the possible cause, treatments, and related literature.

## 2. Case Presentation

**Figure 1:**
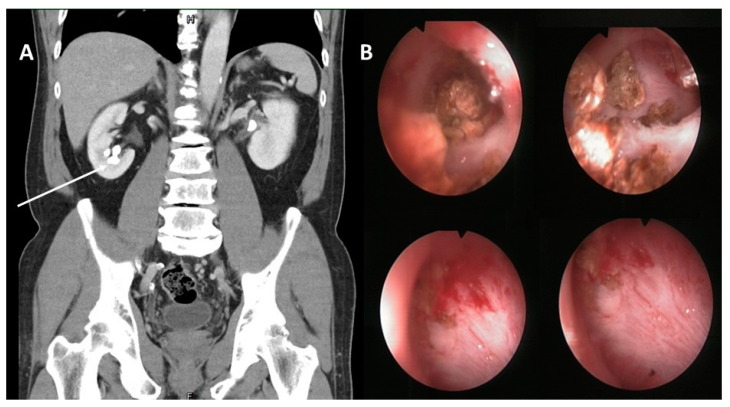
(**A**) Renal stones were noted over the right lower calyx and the tract of the puncture is indicated by a white arrow. (**B**) Renal stones were removed smoothly with slight blood oozing in the renal pelvis at an irrigation pressure of 100 cm H_2_O.

**Figure 2:**
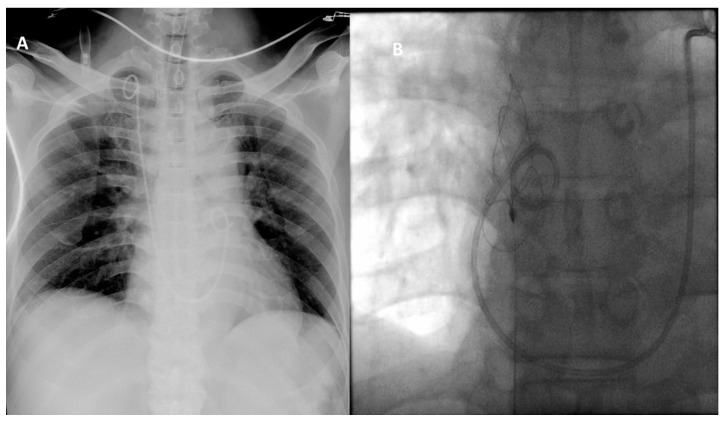
(**A**) The ureteral double-J stent was misplaced into the branch of the renal vein and forward migrated into the inferior vena cava, right atrium, right ventricle, and distal-J end in the pulmonary artery. (**B**) Double-J stent grasped by angiographic snare loop wire via right femoral vein entry access.

A 60-year-old male received one-stage fluoroscopy-free ultrasonography-guided PNL for right renal stones. Under general anesthesia, the patient was placed in a prone position and secured safely to the operative table. No ureteral catheter was placed in advance because many successful puncture experiences were performed without dilatation of the collecting system. A 4.3-MHz convex abdominal transducer (Flex Focus 1202 Ultrasound Scanner, bk medical) was used to localize the stone position and a lower calyx filled with stones was chosen for the puncture (Figure 1A). Under real-time ultrasound monitoring, an 18-gauge 20-cm EchoTip trocar needle (Cook Medical, Bloomington, IN, USA) was advanced into the lower calyx (Figure 1A) and the successful puncture was confirmed by the appearance of clear urine following removal of the obturator. Next, we inserted a terumo^®^ guidewire which slipped from the lower calyx to the renal pelvis. A balloon dilatation catheter (Ultraxx™ Nephrostomy Balloon Catheter, Cook Medical, Bloomington, IN, USA) was further placed along the guidewire to the lower calyx. Ultrasound was performed to confirm the position of the balloon dilatation catheter and saline was injected into the balloon until the water pressure was 20 atm for the radial expansion. After a 5-min maintenance, a 27 Fr. outer sheath was placed along the balloon catheter to the target calyx. Finally, the balloon catheter was removed and the terumo^®^ guidewire stayed in place as a safety consideration. A 24 Fr. nephroscope (RICHARD WOLF Medical Instruments Corporation, Knittlingen, Germany) was used to start the lithotripsy procedure. The procedure was performed smoothly at an irrigation pressure of 100 cm H_2_O with slight blood oozing in the renal pelvis and the renal stones were removed without residual fragments (Figure 1B). Toward the end of surgery, due to the previous terumo^®^ guidewire slipping away from the renal pelvis during the lithotripsy procedure, a new terumo^®^ guidewire was placed without resistance under a nephroscope to a place that looked like the ureterorenal junction and upper ureter. A ureteral double-J stent was inserted through the guidewire under visual inspection. Unexpectedly, a sudden onset of atrial fibrillation with a drop in blood pressure (systolic/diastolic blood pressure: 70/40 mmHg) was noted. In the meantime, we could not find the ureteral double-J stent at the renal pelvis. Emergent X-ray showed the stent had penetrated deeply into the heart (Figure 2A). Due to misplacement following the wrong route into the branch of the renal vein, the stent migrated into the inferior vena cava (IVC) and the distal end was placed into the pulmonary artery. Due to the emergent situation, we did not deploy a new ureteral double-J stent or percutaneous nephrostomy and closed the PNL wound immediately. We consulted an intervention radiologist for the optimal treatment. The ureteral double-J stent was then grasped by the angiographic basket via femoral vein access and was withdrawn (Figure 2B). After surgery, the patient was observed in our intensive care unit that night; no atrial fibrillation or hypotension were noted. The patient’s vital signs were stable and serum electrolytes were within the normal range. He was transferred to the ordinary ward the next morning. The patient was then uneventfully discharged two days later. There was no renal hematoma or hydronephrosis in the clinic follow-up one week later.

## 3. Discussion

The complications of PNL include hemorrhage (1% to 34%), damage to the collecting system injury (2% to 7.2%) or adjuvant organs (0.2% to 4.8%), and other medical issues [5]. However, the intracardiac migration of the ureteral stent has never before been encountered during PNL and delaying the diagnosis might cause mortality.

According to a literature review, there were nine case reports (including ours) of the migration of the ureteral stent into the major cardiovascular system to date, as shown in Table 1 [6,7,8,9,10,11,12,13]. Seven of the events occurred in females and two occurred in males. The age distribution of patients was between 28 and 60 years, with the placement being mainly on the right side, accounting for six. The position of the ureteral stent was in the inferior vena cava (*n* = 5), heart (*n* = 3) and pulmonary vessels (*n* = 1). Ureteral stent insertion via ureteroscopy or cystoscopy was the most common procedure causing this complication. Boari flap, nephrolithotomy, ureterocutaneostomy, and pyelolithotomy were also possible surgeries. To our knowledge, this is the first study presenting such a complication after PNL. The management of ureteral stent withdrawal included cystoscopy, a ureteroscopic approach, percutaneous nephroscopy, endovascular intervention, laparoscopic exploration, and open surgery. The treatment options were dependent on the position of the distal end of the ureteral stent, the condition of the patient, the expertise of the surgeon, and the equipment. Most of the patients recovered uneventfully after ureteral stent retrieval. 

Michalopoulos et al. mentioned that the possible mechanism for the migration of the ureteral double-J stent was erosion of the urinary tract into the vascular system because of the adjacency of the two systems. A contributing factor might be the fragile ureteral wall induced by the stone fragment passage or the stent itself [11]. However, in our case, the etiological mechanism was via direct penetration of the ureter wall, crossing over to the renal vein, migrating into the IVC, and with blood flow-directed movement of the stent, it finally approached the heart and pulmonary vessel. 

The following reasons might be the cause of complication [14]. Firstly, our puncture site might have been too medial or deep, thus the working place was too adjacent to the renal vein; secondly, overzealous dilatation of the renal pelvis/caliceal wall, causing possible injury of the renal pelvis wall; thirdly, concomitant inflammation or infection might have made the renal pelvicaliceal wall more friable; and fourthly, the stent placement was not performed under fluoroscopy monitoring.

However, several studies have already discussed the safety and effectiveness of ultrasound-guided (UG) versus fluoroscopy-guided (FG) PNL. One meta-analysis including 966 patients revealed that UG-PNL had comparable stone-free rates (odds ratio (OR) = 0.95; 95% confidence interval (CI) = 0.67 to 1.35; *p* value = 0.79) but with a lower complication rate (OR = 0.56, 95% CI = 0.36 to 0.86; *p* value = 0.009) [15]. Despite the fact that ultrasonography has emerged as an alternative to fluoroscopy for image-guided PNL, we should always keep in mind that the use of one technique should not exclude the other, and if needed, we should take the combined technique into consideration.

The possible symptoms and complications of intravascular migration of the stent include chest tightness/pain, dyspnea/tachypnea, hypotension, cardiac arrhythmia, myocardial damage, recurrent pericardial effusions, tricuspid valve insufficiency, endocarditis, and embolism to other organs [7]. This case presented with the sudden onset of atrial fibrillation and cardiovascular shock; in addition, the ureteral double-J stent could not be seen under nephroscopy. Emergency chest radiograph revealed the misplacement of the ureteral double-J stent. Finally, the symptoms were relieved after the ureteral double-J stent removal.

Some strategies can be implemented to reduce the risk of this complication. Firstly, the direction of the guidewire should be monitored throughout the entire process either by nephroscopy or fluoroscopy systems. Secondly, low irrigation fluid pressure should be used to prevent excessive intrarenal pressure during the surgery. Thirdly, injury to the medial side of the renal pelvis should be avoided due to the adjacency of the renal vein or IVC. Fourthly, ureteral double-J stent placement should be inserted under fluoroscopic guidance or, if without fluoroscopy, postoperative X-ray should be routinely performed. Early recognition of the migration of the ureteral double-J stent is crucial and it must be removed promptly to avoid the development of more problematic complications.

## 4. Conclusions

We believe this case is the first description of ureteral double-J stent migration into the heart during PNL. Physicians should always be aware of major complications during this procedure and early diagnosis and treatment is emphasized to prevent mortality and morbidity.

## Figures and Tables

**Table 1:** Literature of the migration of the ureteral double-J into the cardiovascular system.

Authors	Sex/Side/Age	Location	Surgery	Catheter Withdraw
Falahatkar et al. [6]	F/L/52	IVC	Ureteroscopy and ureteral double-J placement	Endovascular
Farshi et al. [7]	F/R/28	IVC	Cystoscopy and ureteral double-J placement	Ureteroscopy
Hajji et al. [8]	F/R/33	IVC	Cystoscopy and ureteral double-J placement	Endovascular
Jiang et al. [9]	M/L/53	IVC	Pyelolithotomy	Nephroscope
Maheshwari et al. [10]	F/R/30	IVC	Boari flap	Cystoscopic stent removal
Michalopoulos et al. [11]	F/R/29	Left lung	Nephrolithotomy	Endovascular
Sabnis et al. [12]	F/R/43	Right atrium	Ureteroscopy and ureteral double-J placement	Open surgery
Arts et al. [13]	F/L/57	Right atrium	Ureterocutaneostomy and ureteral single-J placement	Endovascular
Chang et al. (present study)	M/R/60	Right heart	PNL (first-described)	Endovascular

Abbreviation: R, right; L, left; F, female; M, male; IVC, inferior vena cava; and PNL, percutaneous nephrolithotomy. Age is presented as years old.

## Data Availability

The data presented in this study are available on request from the corresponding author.
